# Interoceptive insular cortex participates in sensory processing of gastrointestinal malaise and associated behaviors

**DOI:** 10.1038/s41598-020-78200-w

**Published:** 2020-12-10

**Authors:** Marcelo Aguilar-Rivera, Sanggyun Kim, Todd P. Coleman, Pedro E. Maldonado, Fernando Torrealba

**Affiliations:** 1 Department of Bioengineering, University of California, La Jolla, San Diego, CA 92093 USA; 2 LVIS Korea, Seoul, Korea; 3 Facultad de Medicina, Universidad de Chile, Santiago, Chile; 4 Biomedical Neuroscience Institute, Universidad de Chile, Santiago, Chile; 5 Departamento de Ciencias Fisiológicas, Pontificia Universidad Católica de Chile, Santiago, Chile

**Keywords:** Sensorimotor processing, Neurophysiology

## Abstract

The insular cortex plays a central role in the perception and regulation of bodily needs and emotions. Its modular arrangement, corresponding with different sensory modalities, denotes a complex organization, and reveals it to be a hub that is able to coordinate autonomic and behavioral responses to many types of stimuli. Yet, little is known about the dynamics of its electrical activity at the neuronal level. We recorded single neurons in behaving rats from the posterior insula cortex (pIC), a subdivision considered as a primary interoceptive cortex, during gastrointestinal (GI) malaise, a state akin to the emotion of disgust in humans. We found that a large proportion of pIC neurons were modulated during the rodent compensatory behaviors of lying on belly (LOB) and Pica. Furthermore, we demonstrated that LOB was correlated with low-frequency oscillations in the field potentials and spikes at the theta (8 Hz) band, and that low-frequency electrical microstimulation of pIC elicited LOB and Pica. These findings demonstrate that pIC neurons play a critical role in GI malaise perception, and that the pIC influences the expression of behaviors that alleviate GI malaise. Our model provides an accessible approach at the single cell level to study innate emotional behaviors, currently elusive in humans.

## Introduction

Sensations, such as the sickness we feel after eating spoiled food, or the feeling of butterflies when we are in love, are poorly understood. Such diffuse sensations, which characterize a large variety of emotions, are assumed to be related to the activity of the insular cortex (IC), which receives information from interoceptors distributed all over the body^1^. Functional MRI studies have found overlapping co-activation of different insular subdivisions during tasks involving affective processing, sensorimotor processing, high-level perception and attention^2^. In rats, it has also been demonstrated that this cortex is a key structure in the perception of bodily needs that provide direction for motivated behaviors^3^.

The mammalian IC is the highest brain structure in the interoceptive pathway^1,4,5^. It processes signals from dozens of interoceptors as well as other parts of the brain^1,6–8^. In rodents, the IC has been shown to be a complex cortical structure with several anatomical and functional subdivisions^4,9–11^.

The granular pIC region receives inputs directly from the interoceptive thalamus and has been considered a primary interoceptive cortex^12^.

Previous electrophysiological (ephys) studies of the interoceptive pIC have involved anesthetized animals and have shown that neural activity in the pIC changes in response to stimulation of tongue thermoreceptors^13^, arterial chemoreceptors^4,5^ and baroreceptors^4,14^, as well as the vagus nerve^8^. However, outside the anesthetized setting, many questions remain about the extent to which specific neurons that modulate their activity to interoceptive stimuli could also drive behaviors indicative of GI malaise and relief.

Considering the key anatomical location of the pIC as the site immediately downstream of the interoceptive thalamus and upstream of executive cortices, we hypothesize that its neuronal activity represents the perception of GI discomfort and that it is instrumental in triggering behavioral relief. Thus, we recorded pIC neural activity before and after inducing GI malaise by injecting intraperitoneally (IP) LiCl, a well-characterized poison that induces GI malaise and toxicosis in rats^15,16^ and typically causes LOB and Pica behaviors^16,17^. In the absence of LiCl injection, we applied electric stimulation through the recording electrodes in the pIC to explore the behavioral consequence of its activation.

## Results

### Behaviors expressing GI malaise induced by systemic LiCl administration

**Figure 1:**
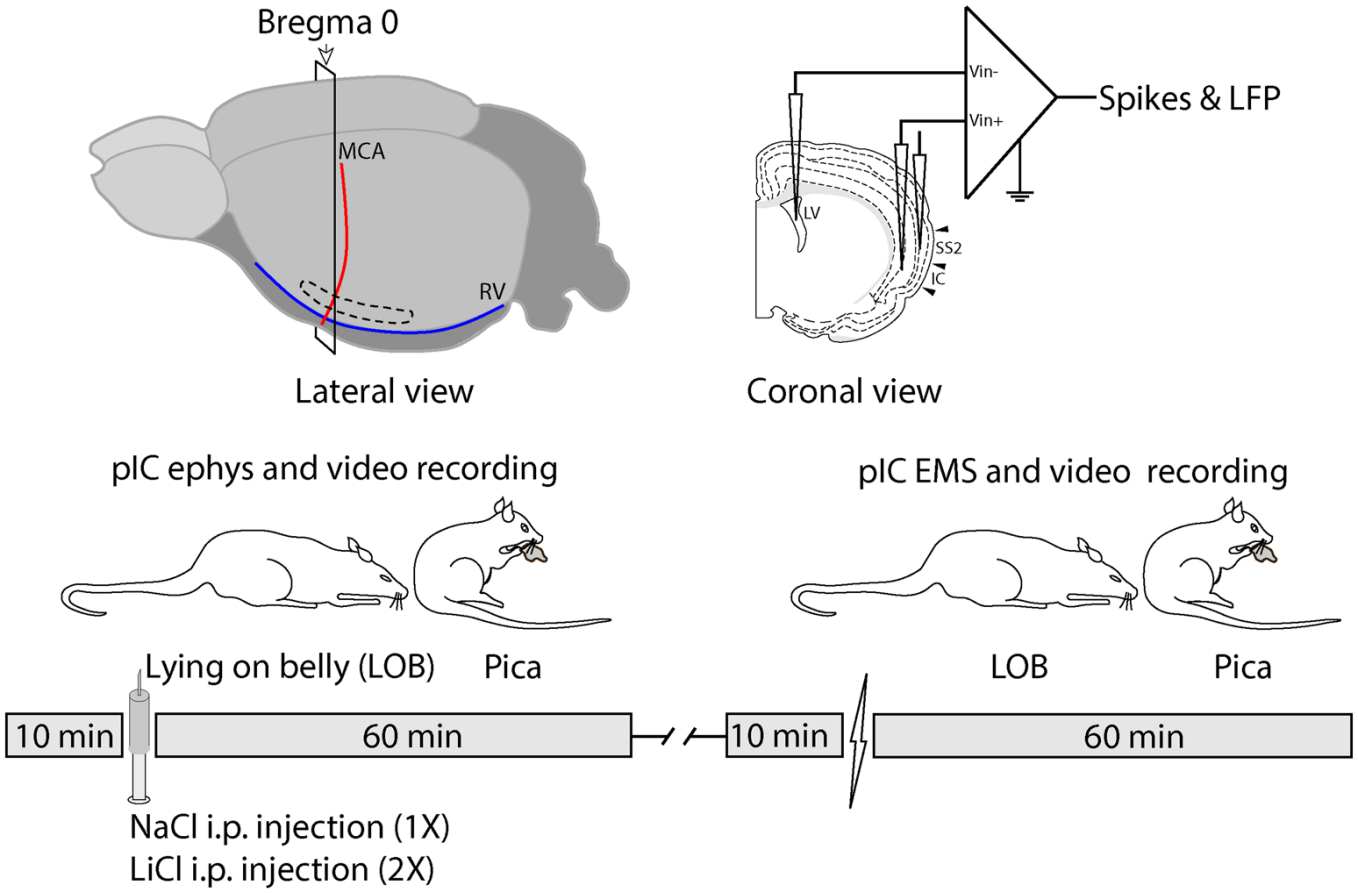
To study how the activity of single neurons in the pIC represents GI malaise, we administered LiCl IP and then recorded the neural activity while simultaneously video-recording LOB and Pica as the behavioral signs of GI malaise. To examine how specifically the neural activity in the pIC responds to GI malaise, we compared these results with those obtained using NaCl IP. To determine how specifically the pIC responds to GI malaise, the activity of somatosensory cortex neurons was also compared to that of the pIC during LiCl-induced malaise. To explore the behavioral consequence of artificial pIC activation we used EMS in the absence of LiCl injection.

**Figure 2:**
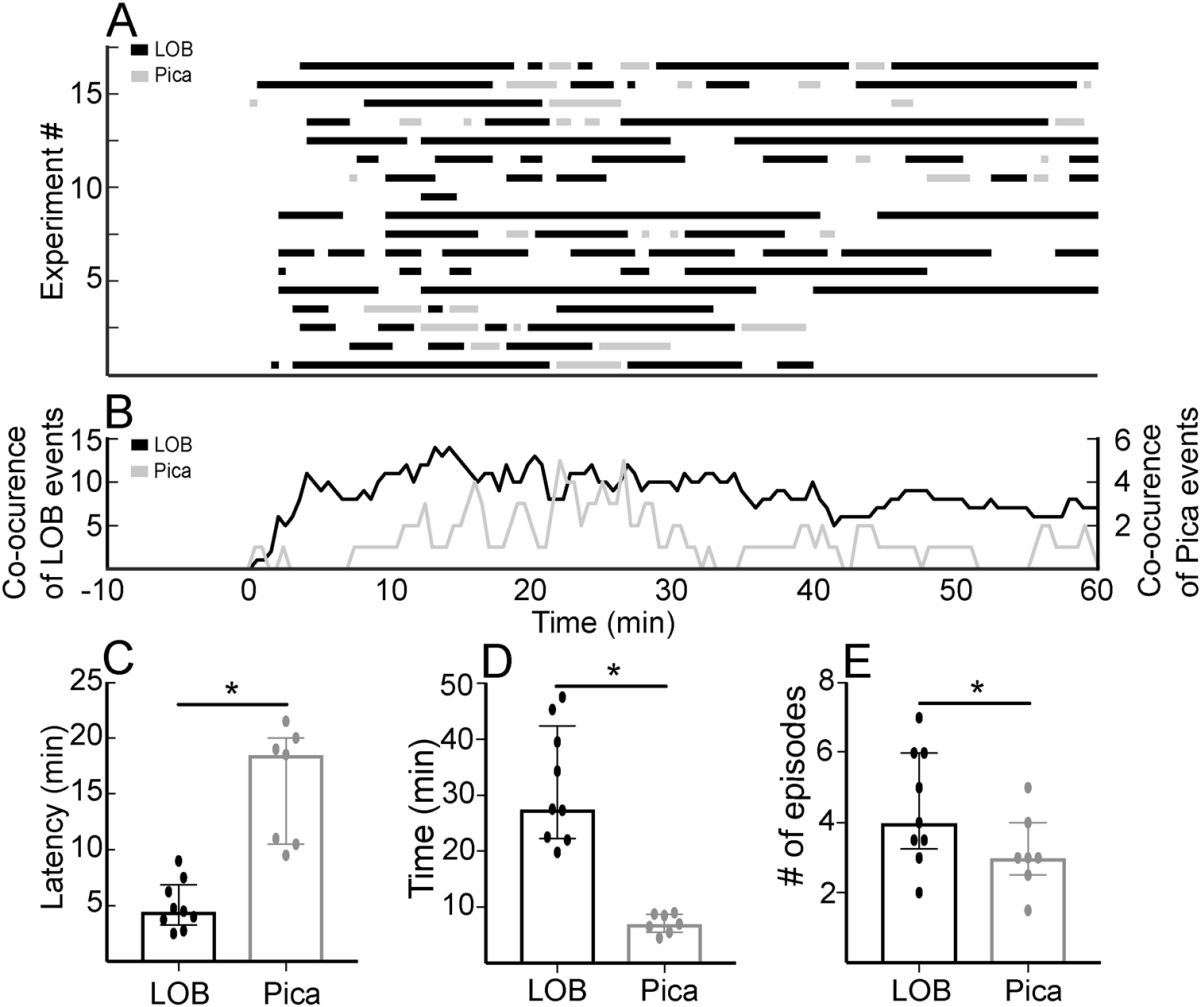
Quantification of behavioral events that express gastrointestinal malaise. (**A**) Graphical representation of LOB and Pica episodes after IP LiCl injection at time 0 for all the experiments. (**B**) Index of co-ocurrence of behaviors estimated as the sum of events throughout the experiments. The black and gray lines represent the cumulative LOB and Pica episodes, respectively. (**C**) Latency comparison for the first LOB and first Pica episodes after LiCl administration (asterisks, *p* < 0.05, Mann–Whitney test). (**D**–**E**) Statistical description of the behavioral expression of GI malaise. Bars represent median, whiskers the interquartile range, and asterisks a *p* value < 0.05 on the Mann–Whitney test.

LOB and Pica (Fig. 1) were not observed in any of the animals before IP LiCl injection (Fig. 2), but after LiCl administration all the nine rats showed GI malaise expressed as LOB. The temporal course and frequency of LOB and Pica are depicted by Figs. 2A–E. The median latency to the first LOB episode was 4.5 min and the duration was 27.5 min, with a median of 4 episodes per animal. Pica was observed in seven out of the nine rats, with a median latency of 18.5 min and a duration of 7 min. Pica behavior was consistently observed both after and between LOBs with a median of 3 episodes per rat. We observed differences in the time between both behaviors (Fig. 2A–D). LOB onset occurred before Pica (Mann–Whitney test; U = 0, *p* < 0.05) and this behavior was four times longer than Pica (Mann–Whitney test; U = 0, *p* < 0.05). We observed a bigger number of LOB episodes in comparison to Pica (Mann–Whitney test; U = 15.5, *p* = 0.0476).

### Firing rate of pIC neurons after IP LiCl injection

**Figure 3:**
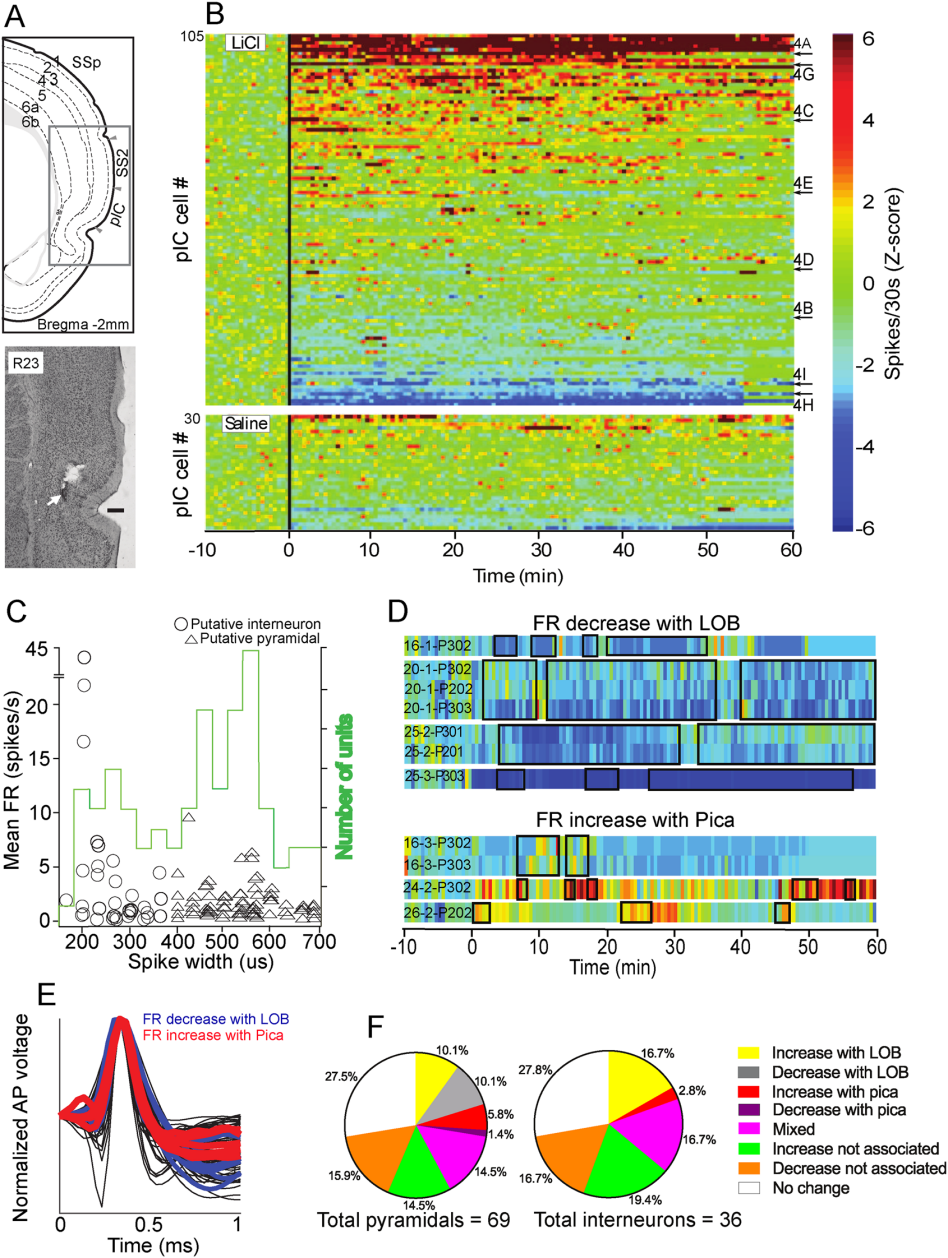
pIC neurons responded to LiCl injections and GI malaise. (**A**) Top panel depicts an anatomical drawing adapted from^47^ . pIC, posterior insular cortex; SSs secondary somatosensory cortex. Bottom panel shows an electrolytic lesion (white arrow) in the pIC, 0.3 mm ventral (scale bar) to the recording site. (**B**) Top, colored PSTH for cells responsive to IP LiCl injection (at time 0). The arrows and letters denote the position in this plot of the units shown in Fig. 4. Bottom, subset of neurons showing responses to IP NaCl injection. The color codes represent the intensity of firing rate. (**C**) Scatter plot depicting the spike peak-to-trough duration and firing rate for all the pIC neurons. Pie charts show proportions of pIC cell types responding to LiCl injections and behaviors. (**D**) PSTH shows the changes in single putative pyramidal neurons activity during LOB (top) and Pica (bottom). Black rectangles on the PSTH represent LOB and Pica on the top and bottom chart respectively. The firing rate is depicted with the same color code as in (**B**). (**E**) Waveform of the same putative pyramidal neurons as in (**D**), whose activity was associated with LOB and Pica. (**F**) Pie charts showing the proportions of putative pyramidals and interneurons associated with LOB and Pica.

We recorded 105 total pIC units (Fig. 3A,B) from the nine rats described above and examined the modulation of their electrical activity induced by LiCl injections. Only four of these neurons were classified as multiunit (MU), while the remaining neurons were classified as single units (see material and method). The basal median FR (firing rate) estimated from all the neurons was 0.6 spikes/sec with a range of 0.1 to 43.6 spikes/sec (Fig. 3C), consistent with previous findings^18^. We found that 71% of the recorded units (76/105) changed their FR after systemic LiCl administration (permutation test, *p* < 0.01). Figure 3B shows the responses of all recorded pIC neurons sorted by the average FR post-injection. No differences in the FR magnitude of pIC neurons in response to the first and second IP LiCl injection were found.

We further examined the spike width average of each neuron to better understand how pIC cell classes participate in the behaviors of LOB and Pica. The distribution of waveform widths was bimodal, suggesting two distinct neural populations (Fig. 3C; Hartigan’s dip test, D = 0.0482; *p* = 0.047). We considered those cells whose action potential peak-to-trough was less than the limit of 396 μs (K-means test) to be putative interneurons. The median waveform width of these spikes was 264 μs (N = 36), while putative pyramidal neurons (N = 69) had a wider spike waveform with a median width of 528 μs (Mann–Whitney test; U = 0, *p* < 0.0001). We found heterogeneity in FR in the population of putative interneurons, similar to that reported for the orbitofrontal cortex (Fig. 3C), a region considered to be an extension of the rostral agranular insula^19,20^. More importantly, all of the neurons that showed a decrease in their FR in conjunction with LOB, and all but one neuron that increased their activity during Pica epochs, had a long peak-to-trough (Fig. 3D–E). A common feature of these putative pyramidal neurons was low basal FR, with a median of 0.3 spikes/sec and 0.1 spikes/sec for those with a decrement in activity during LOB and an increment in FR associated with Pica respectively. The remaining behavior associated response types, had a similar proportion of putative pyramidal cells and interneurons (Fig. 3F).

Most of the putative pyramidal neurons that showed activity associated with LOB and Pica were recorded from deeper layers. However, we did not find a spatial grouping of neurons with similar responses to GI malaise or its relief in the rostral-caudal axis of the pIC (Figure S4). Overall, these results demonstrate that single unit activity in the pIC would have noteworthy specificity to LOB and Pica.

**Figure 4:**
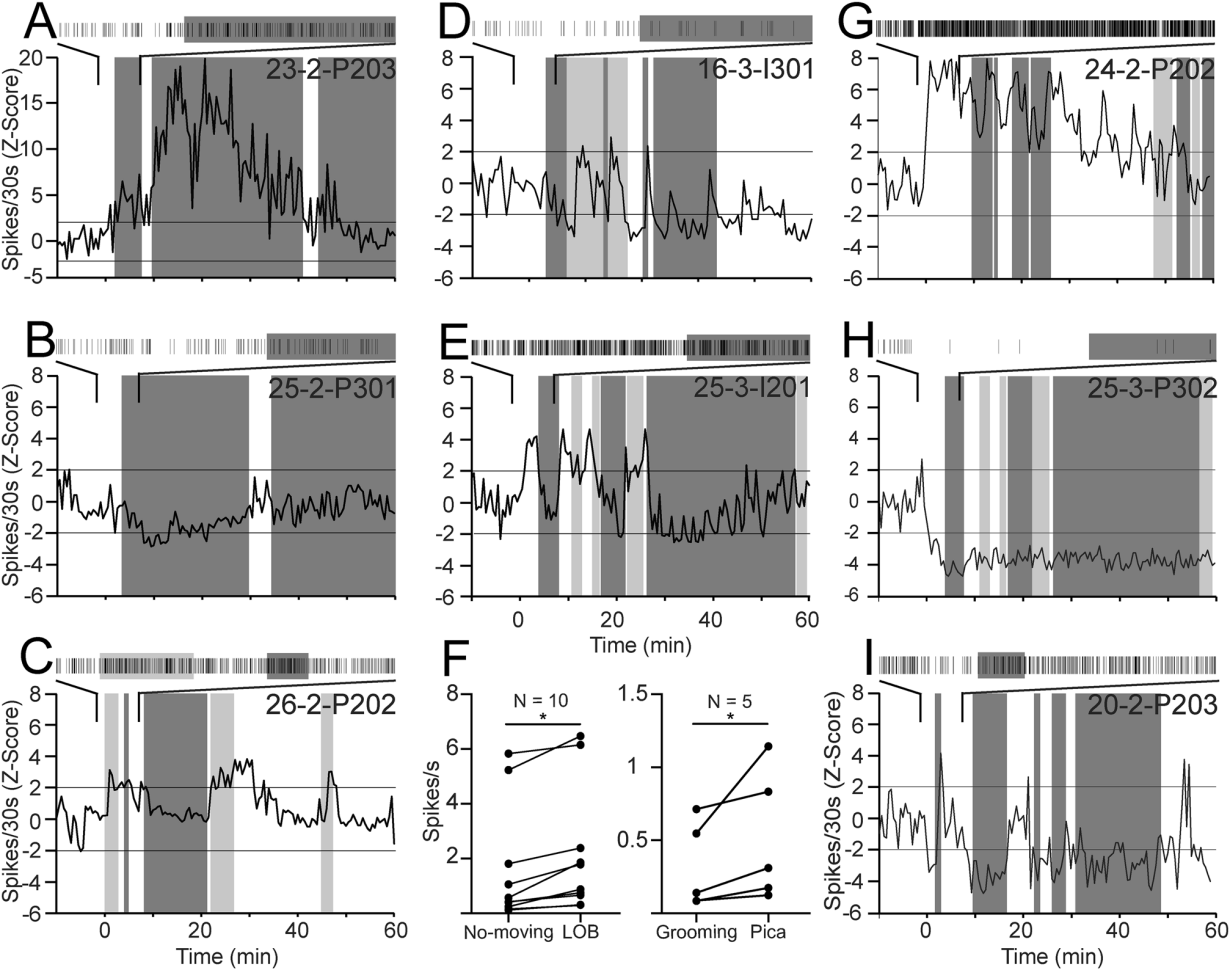
Discharge patterns of pIC neurons associated with LOB and Pica. (**A**–**B**) Example of putative pyramidal neurons that increased and decreased their activity during LOB (dark gray shadow). The raster plots above each graph expand the section indicated by the lines. (**C**) Putative pyramidal cell that increased its activity during Pica (light gray shadow). (**D**, **E**) are examples of putative interneurons with a mixed response that involved a decrease of activity during LOB, and an increase during LOB-off and Pica. Cell E also showed an increment immediately after IP LiCl injection. (**F**) Averaged activity of pIC neurons responding to the perception of GI malaise triggered by IP LiCl administration and its relief. The asterisk depicts *p* < 0.05 on Wilcoxon test; bars represent mean and standard deviation. (**G**–**I**) Examples of putative pyramidal neurons that exhibited a change in their firing rate immediately after IP LiCl injection until the end of the recording. Horizontal lines depict −/ + 2 standard deviations.

In this regard, twelve percent of the pIC neurons (13/105; 5 rats) increased their activity during LOB epochs, as shown in Fig. 4A. This recording was made from a putative pyramidal neuron whose FR remained high during the first two LOBs (dark gray shadow) but transiently increased its FR during the third episode. On the other hand, six percent of neurons (7/105; 3 rats) decreased their FR during LOB epochs (Fig. 4B). Interestingly, for some neurons, the change in FR was associated with the absence of overt GI malaise, as they seem to be coding relief rather than malaise. Thus, almost 5% neurons increased their activity after LOBs or between them, and this rise coincided with Pica (light gray shadow; 5/105 neurons; 4 rats; Fig. 4C). Also, fifteen percent of neurons (16/105; 8 rats) showed mixed responses to LiCl-evoked behaviors. Seven of them displayed an increase in activity between LOBs during Pica, but a reduction during LOB. Two of these neurons that showed mixed responses were MU. The remaining neurons showed an early increment in FR immediately after IP LiCl injection, in addition to the increase that occurred between LOBs or immediately after a LOB episode, but not necessarily coincident with Pica (Fig. 4D–E).

To rule out that FR modulations were a direct consequence of a decrease in locomotion, we compared the activity of neurons whose FR increased significantly with LOB with the baseline recording while the rat was not moving (Fig. 4F). We found that these neurons had higher activity during LOB (Wilcoxon test; W = 55, *p* < 0.001), indicating that they responded to GI malaise expressed as LOB and not simply the absence of movement. However, the decrement in FR during LOB was not lower than the observed during the not moving periods from baseline. In addition, we compared the FR of neurons whose activity increased during Pica against the baseline (Fig. 4F) when the rat was grooming (a behavior that involves the oral cavity) and found these neurons had a higher FR during Pica (Wilcoxon test; W = 15, *p* < 0.0312). This result suggests that some pIC neurons respond to the perception of GI malaise relief rather than to effector mechanisms associated with Pica.

Finally, nearly a third of the total neurons recorded in the pIC (34/105) changed their FRs in close temporal proximity to the IP LiCl injection, but not associated with LOB or Pica (Fig. 3F). Half of these neurons showed a sustained increase of their FR, and in some of them this increase occurred immediately after LiCl administration (Fig. 4G). One of these neurons was classified as MU. The other half decreased their activity immediately after IP LiCl injection. For some of them, this decrease was sustained (Fig. 4H), while other neurons displayed brief overshoots of their activity in between LOB episodes (Fig. 4I). One of these neurons was classified as MU.

Overall, these results indicate that the neural activity in the pIC appears to be involved both in the perception of GI malaise and in the initiation of Pica to alleviate such malaise. Given the low number of neurons recorded in this study, we acknowledge as preliminary results the proportions of neurons showing the firing characteristics associated with LOB and Pica.

### Firing rate modulation by NaCl versus LiCl injections

The relationship between FR in pIC and GI malaise is further supported by the comparison between the effects of sodium versus lithium (Fig. 3B and Table S1). We compared the neural activity in five out of nine rats recorded from the same sites after IP NaCl injection and then after a dose of IP LiCl, with a delay between injections of 48 h. We found a higher proportion of neurons that increased their FR coincident with LOB after IP LiCl injection, and only one neuron that showed an increase in activity during a single and short LOB period after NaCl administration (Fisher exact test; *p* = 0.019). Regarding the neurons that increased their activity unassociated with LOB after IP NaCl injection, mostly the increments occurred immediately after the injection; this could be explained by distress triggered by the manipulation/injection. The same observation was made for the neurons that decreased their firing after IP NaCl injection. Similar results were found for a small proportion of secondary somatosensory cortex (SS2) neurons (see supplementary results).

### Oscillatory activity of the pIC

**Figure 5:**
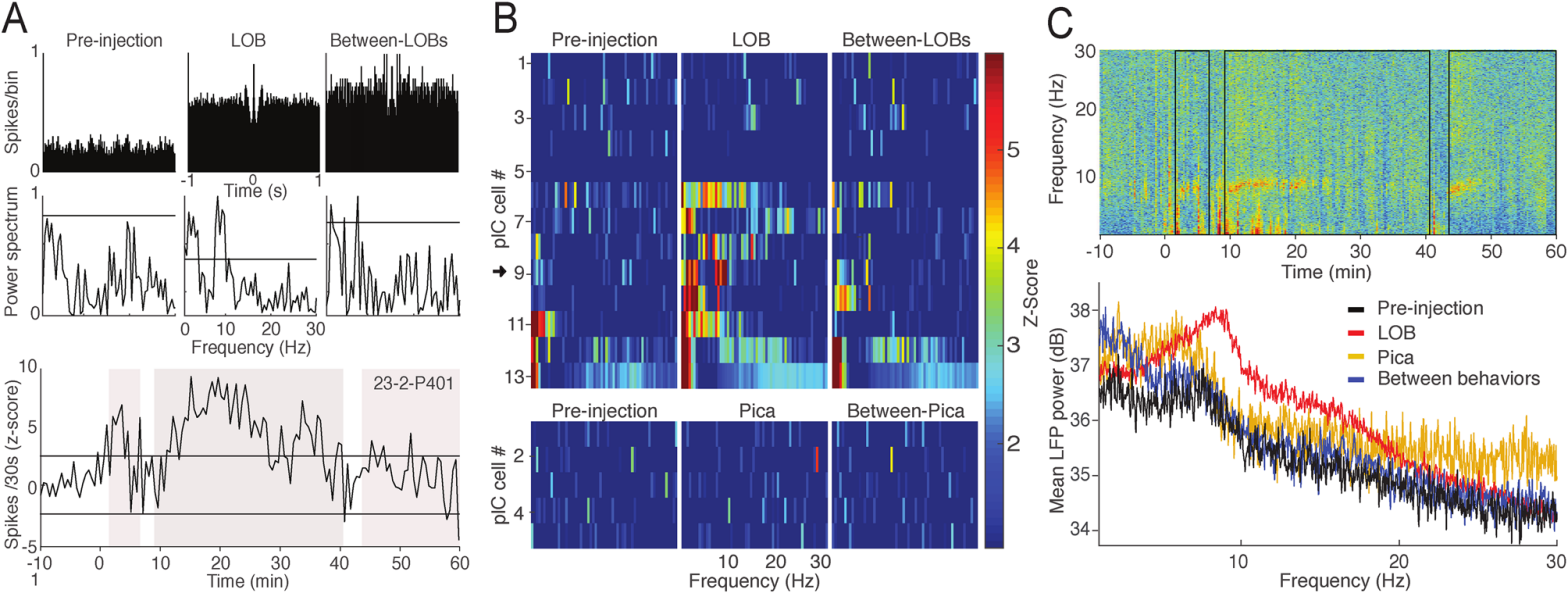
Low frequency activity in spikes and field potential associated with LOB. (**A**) Normalized auto-correlograms and power spectrum plots for pre-injection, LOB and between-LOB periods (not including Pica epochs) of the PSTH shown below, where the shaded regions depict three LOB periods. Horizontal line on the power spectrum plots represents two standard deviations from the averaged magnitude. (**B**) Spectrograms for all thirteen and five neurons whose firing rate increased significantly during LOB and Pica, respectively. (**C**) Spectrogram of LFP signal from one channel of a tetrode used to record single units, showing activity associated with LOB (same experiment shown in **A**). The rectangles enclosed by black lines depict the three LOB episodes shown in (**A**) Below, the averaged power spectrum of five LFP signals recorded in the pIC of four rats. Between-LOB periods do not include Pica epochs.

Because oscillatory activity plays an important role in establishing functional connectivity in the cortex, we examined and characterized this activity in our recordings from the pIC. We also aimed to identify the prominent oscillatory activity in this cortex to be later used as a pattern for our EMS studies. In both spike and local field potential (LFP) recordings, a predominant activity in the low frequency band with a peak at theta (8 Hz) were seen during LOB. Figure 5A shows auto-correlograms and their respective power spectrums for different periods from the FR of the neuron shown at the bottom of this figure. It is possible to see a predominant peak of oscillatory activity over two standard deviations at ± 125 ms when the rat was in LOB. The spectrogram for each of the thirteen neurons that showed an increase in their FR during LOB and for the five neurons excited during Pica was computed (Fig. 5B). We found that while many neurons displayed a prominent oscillatory activity under 10 Hz during LOB, and some of them in between LOB epochs (that did not consider Pica episodes), none of the neurons that increased their FR during Pica showed oscillatory activity associated with this behavior. Importantly, all of the neurons that showed an increase of power under 10 Hz during LOB were recorded from electrodes placed in the posterior IC from − 0.1 to − 2 mm from bregma. In Fig. 5C, we show the spectrogram of one field potential signal, when the rat showed three LOB periods (same experiment shown in A). A sustained increment in theta power immediately after LiCl injection was observed, coinciding with the onset of the three LOB periods. Transient increments in lower frequencies were also detected. The averaged spectral profiles of LFPs recorded from multiple experiments showed a prominent peak around 8 Hz for LOB epochs.

### Electrical microstimulation of the pIC induces LOB and Pica.

**Figure 6:**
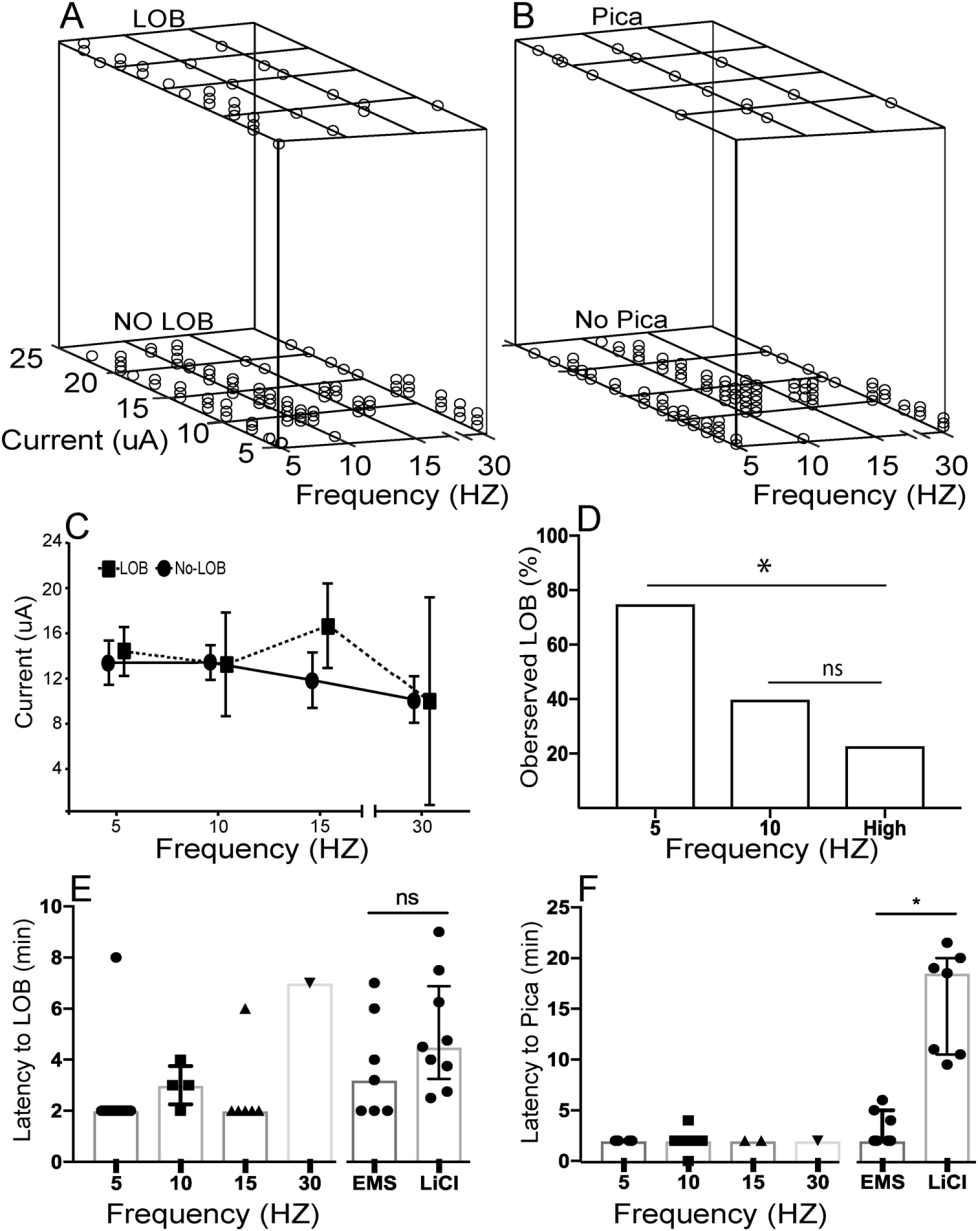
Electrical stimulation of the pIC at low frequencies triggered LOB and Pica. (**A**–**B**) Scatter-plots of behavioral outcome considering the whole set of trials in relation to frequency and current levels of EMS. In both plots, the value 1 in the Z-axis represents that LOB or Pica was triggered by stimulation of the pIC. (**C**) Magnitude of current necessary to trigger LOB at different frequencies of EMS. Vertical bars denote 0.95 confidence intervals (**D**) Summary of behavioral outcome for the total number of sessions at different frequency of EMS. (**E**–**F**) Latency to LOB and Pica onset at different frequencies per each experiment. Then a latency to LOB and Pica comparison between EMS (all frequencies per animal) and IP LiCl injection. Bars represent median and whiskers the interquartile range.

To examine whether direct activation of pIC could trigger behaviors to alleviate malaise, we applied EMS at different levels of the rostro-caudal axis in the granular region of the pIC in six out of the nine rats from which spikes and LFP were recorded. In addition, we applied EMS in the pIC of two more rats not injected with lithium. A stimulation trial, consisting of a train of pulses for a specific level of current and frequency, was applied through two wires of tetrodes used to record pIC neural activity previously during lithium experiments. We found that trains of pulses at 5, 10, 15 and 30 Hz triggered LOB and Pica in seven out of eight (88%) rats, one of them being naive for LiCl. Using the whole set of trials (N = 121; 8 rats) we performed a logistic regression analysis (Fig. 6A) and found frequency had an inverse correlation to LOB (Wald’s X^2^ = 5,655; *p* = 0.017). Such correlation was not observed with Pica (Fig. 5B; Wald’s X^2^ = 0.066; *p* = 0.796). There was no difference in the intensity of current that triggered or failed to trigger LOB (DF = 1; F = 0.838; *p* = 0.361), in the intensity of current among the frequency levels (DF = 3; F = 0.914; *p* = 0.436), or the interaction of both variables (DF = 3; F = 0.951; *p* = 0.418) evaluated through a two-way ANOVA (Fig. 6C). This suggests that frequency is the main feature of EMS that explains the expression of LOB by artificial activation of the pIC. When we estimated the proportion of sessions at each frequency in which LOB occurred (see Methods), a greater proportion of LOB at 5 Hz was observed than at higher frequencies (15 and 30 Hz) of stimulation (Fisher exact test; *p* = 0.0294). LOB behavior was triggered in 75% of the sessions (6/8; 4 rats) at 5 Hz, 40% of sessions (4/10; 4 rats) at 10 Hz, but in just 23% of the sessions (3/13; 3 rats) at high frequency (Fig. 6D). We observed 19% (6/31) of the time that both behaviors were triggered by low-frequency (theta: 5–10 Hz) of stimulation during the same session in six out of the eight rats. The latency to LOB triggered by EMS was similar to that triggered by LiCl (Fig. 6E), and LOB rarely extended longer than a few seconds after EMS offset. On the other hand, the latency to Pica triggered by EMS was shorter than that by LiCl, and it lasted only during stimulation (Fig. 6F).

Stimulation of SS2 did not trigger LOB or Pica in two additional rats from which we recorded SS2 neurons after IP LiCl injection (supplementary Fig. 2), nor was Pica or LOB triggered through the electrode placed in the lateral left ventricle in the ten rats used in the electrical stimulation experiment.

We did not find evidence of a particular locus for LOB and Pica in the rostral-caudal axis of the pIC triggered by EMS (supplementary Fig. 5).

## Discussion

There is evidence that autonomic and behavioral responses to toxicosis triggered by lithium require the integrity of lower levels of the interoceptive pathway. We now speculate on what role the pIC plays in LOB and Pica, two behaviors that probably have been key for the fitness of non-emetic omnivore species such as the rat. First, a common interpretation of LOB is that this behavior represents a postural index of GI malaise^3,21^, while Pica is considered an illness-response behavior acting to mitigate the effect of toxic agents^16,22^. Secondly, the pIC has been considered a primary sensory cortex based on anatomical criteria developed for other sensorial modalities. However, in light of our findings, the pIC appears to be processing more than only primary interoceptive information. Rather, it could drive behaviors corresponding with emotions and bodily needs.

We found that pIC neurons represent sensory activity related to abdominal pain, GI discomfort^3,23–26^ and relief from both afflictions following a GI discomfort period. These “relief” neurons responded between LOBs, coincident with Pica, as well as at LOB offsets, when Pica was not present. Some pIC neurons that increased their FR in close temporal proximity to the IP LiCl injection showed a sustained increase in activity almost until the end of the recording. This suggests that some pIC neurons also encode a generalized level of GI malaise, triggered by the effect of lithium at different levels of the interoceptive pathway. It has been demonstrated in anesthetized rats that an IP injection of LiCl elicits a tonic FR increase in both the parasympathetic and sympathetic visceral afferents, with a timing similar to that reported here^26^. At the same time, we cannot unequivocally relate GI malaise perception or its relief to the mixed responses to LOB or Pica shown by some pIC neurons. This is because systemic lithium may induce a variety of adaptive reactions in rats, such as drooped eyelids and piloerection, which may be signs of abdominal pain^27^, as well as hypothermia, delayed stomach emptying^21^, and a decrease in respiratory frequency^28^.

In contrast with LiCl injections, IP NaCl delivery resulted in much smaller changes in the FR of pIC neurons. These small changes in the activity of pIC neurons after IP NaCl injection could be explained by the low temperature of the solution being infused into the peritoneal cavity; by abdominal pain triggered by the injection; or because the hepatic portal osmotic and sodium receptors were activated, and they project to the insula^29^.

Our findings also suggest that injection distress may be represented not only in pIC but also in SS2. Both regions, known to receive nociceptive inputs, had neurons that increased their FR after injection. Further, some of these neurons kept a high FR between LOB periods in some cases coincident with Pica, implying that they may also represent GI relief. However, the relief neurons were found nearly exclusively in the pIC.

One methodological limitation in our study is that while a change in FR can easily be detected during off-line analysis, the exact onset and offset of LOB behavior cannot be detected with the same precision. Nonetheless, it was still possible to identify a clear association between changes in spike rates and this behavior, on the scale of seconds. With regards to Pica, it was observed almost exclusively after LOB, likely when the rats felt well enough to move about, pick up wood shavings, and chew or eat them.

The perception of GI malaise likely originates from the activation of a number of peripheral and central receptors in the interoceptive pathway, which implies an important degree of interoceptive information convergence about different physiological parameters onto single pIC neurons. A large portion of neurons in the nucleus of the solitary tract (NST) receives excitatory input from pharyngoesophageal and peripheral chemoreceptors, pulmonary vagal c-fiber, and cardiac receptors^30,31^. At the cortical level, there is evidence of convergence where more than half of pIC neurons responded to more than one interoceptive stimulus^4^. Importantly, the inactivation of the pIC prior to IP LiCl injection attenuates the expression of LOB^3^. On the other hand, in monkeys the activation of the anterior insula by electrical stimulation evoked food refusal and retching^32^. In humans, such stimulation of the posterior insula triggered a variety of abdominal sensations, while activation of the anterior insula triggered gastric motility^33^.

LFP oscillatory activity in the theta band during LOB could reflect the convergence on pIC neurons of diverse visceral inputs from the interoceptive thalamus. Alternatively, such oscillations could reflect the synchronization of the interoceptive IC with other cortices^34^. Saito et al., showed theta oscillations in response to capsaicin in pyramidal neurons in layer V of the interoceptive IC, a location where cortical output is sent to both frontal cortical and subcortical brain areas. In our work, most of the neurons that showed theta activity during LOB were classified as putative pyramidal neurons recorded from the deeper layers. Interestingly, most of the evidence suggests that theta is associated with exploration, for instance sniffing is temporally related with hippocampal theta rhythm^35^, as well theta is reflecting hippocampal and prefrontal-directed strategic control during exploratory choices^36,37^; however, rats do not explore during LOB a behavioral proxy of GI malaise. In addition, LiCl reduces high frequency breathing, a main component of sniffing^28^. Thus, it is highly probable that pIC oscillatory activity in the theta band during LOB is signaling GI dysregulation, encompass by changes in different physiological parameters. An increased theta power in frontal-posterior, parietal and temporal brain regions has been reported in patients with anorexia nervosa^38^. In rats, LiCl triggers anorexia, but promotes the consumption of non-nutritive substances during Pica. It has been suggested that the interoceptive insula has a central role in anorexia nervosa^39,40^. Our study may contribute to understand the relationship between GI malaise and eating disorders by clarifying the neuronal activity present in the insula during GI discomfort.

Finally, it has been shown that the frequency of licking ranges between 5 and 10 Hz, and gustatory insula neurons follow such oscillations^41^. However, we found that Pica neurons in the interoceptive pIC do not show oscillatory activity in the theta band, dismissing the interpretation that these neurons are representing effector mechanisms associated with Pica. Rather, Pica neurons may be signaling relief from GI malaise triggered by lithium.

The importance of pIC neuronal activity for the expression of GI malaise and its relief is further supported by electrical microstimulation of the pIC, which resulted in LOB and Pica behaviors similar to how the rats responded to lithium injections. A low current of tens of microamps was applied between two wires of the same tetrode in the pIC, with a few tens of microns between wires. It is highly likely that the perturbation was restricted to a small volume of neural tissue, directly activating a few hundred neurons. Also, it could indirectly affect synaptic release, causing post synaptic neurons to fire^42^. Thus, it is probable that interoceptive information flows from the pIC to the rostral agranular insula and then to more frontal cortices to drive compensatory behaviors. It has been reported that waves of voltage propagate from posterior to anterior cortical regions after stimulation^43^. It is also possible that the pIC drives alleviating behaviors through the posterior agranular insula and then to the striatum, a brain area well known to underlie motivated behaviors.

Interestingly, the electrical stimulation of the pIC in the rats naive for lithium also triggered LOB and Pica. This suggests that the pIC could store bodily representations that coordinate different cortical and subcortical areas to drive behaviors related to GI discomfort. We note that in two out of seven rats, Pica happened before LOB. Also, we observed that stimulation triggered low-frequency breathing, drooped eyelids, and piloerection before rats expressed LOB and Pica. This could imply that electrical stimulation may trigger top-down bodily changes through neurons that project to the amygdala and the medial prefrontal cortex, two structures with autonomic control. Such bodily changes could be represented by pIC neurons in a bottom-up fashion, and then trigger LOB and Pica.

Overall, our results indicate that pIC activity is necessary for the behavioral expression of GI malaise, a state akin to the negative emotion of disgust in humans. The importance of the insula in the perception of malaise has previously been demonstrated in rodents^3^, monkeys^32^, and humans^44^. For example, in humans, electrical stimulation of the insula induces a variety of bodily sensations, many of them related to ingestive behaviors, GI related perceptions, and the emotion of disgust^33,45,46^. The present results extend those findings by showing that the pIC may also drive homeostatic compensatory behaviors such as LOB and Pica. Our results complement those described for emetic species such as monkeys and humans, linking insula activity with the GI system and disgust. In his pioneering work, Penfield showed that electrical activation of the human insula triggers not only the perception of different bodily states and feelings, but also motor patterns involving the ingestive system. Recently, Rizzolatti’s work^32^ extends these findings to non-human primates, showing a detailed differentiation in the rostro-caudal axis of the insula that reveals a sensorimotor role for the posterior insula. In light of these seminal works, our experiments in behaving rats, a mammalian species widely used to study the neural basis of complex behaviors, further contribute to understanding of the pIC by providing evidence about the role of pIC activity on bodily needs and emotions.

## Methods

### Surgery and recordings

All experimental procedures were approved by the Institutional Animal Care and Use Committee of the Faculty of Medicine, Universidad de Chile and the Faculty of Biology, Pontificia Universidad Católica de Chile. In addition, the National Institute of Health guidelines were followed in accordance with protocols stated in publication No. 94–3207. Before surgery, adult male Sprague–Dawley rats, were anesthetized with ketamine (12 mg/kg i.p) and acepromazine (1 mg/kg i.p). A linear array of four tetrodes (12 µm NiCr wire; impedance of 1.5 MΩ @ 1 kHz), mounted in a custom manipulator, was implanted 6 mm lateral to the midline and centered at bregma in the rostral-caudal axis^47^. The distance between tetrodes was ~ 350 μm. A reference tetrode was implanted in the lateral ventricle at bregma − 0.5 mm, lateral 2.0 mm and 4 mm in depth. After surgery, the rats received ketoprofene (1 mg/kg im) and enroflaxine (5 mg/kg im) for three and five days, respectively. In the days following surgery, the tetrodes were moved down 600 µm per day until reaching a depth of ~ 4.7 mm.

In each session, only three tetrodes were recorded simultaneously because of the amplifier’s channel capacity. The signals were amplified (by a factor of 10,000), band pass filtered for single unit activity and LFP, sampled at 30 kHz, and then stored for off-line analysis. Extracellular waveforms that exceeded a voltage threshold above two standard deviations from the average noise were extracted. Waveforms were then sorted (LabWindowsTM/CVI software) using two-dimensional plots of peak to peak and principal component of spike waveforms. Using these sorted spikes, we reconstructed the spike trains across time. Spike clusters for which more than 1% of the inter-spike interval was less than 3 ms were declared multiunit, otherwise we referred to them as single units^48^. We recorded an average of twelves neurons per rat.

### Behavior and IP LiCl injections

On average, we performed three sessions of recordings in each rat. First, we recorded the behavior and single unit activity for 10 min before (baseline) and 60 min after an IP injection of NaCl (0.9%; 5 ml/kg), which should have a minimal effect on cell activity. Then, after 48 h, we recorded single neural activity and behavior after an IP LiCl injection (32 mg/kg; 0.15 M; 5 ml/kg) without moving the electrodes. We employed this low dose of LiCl in order to prevent an extensive amount of time of the rats in LOB, thus hindering the expression of Pica^3^. When engaged in LOB, the rats lie with their bellies flattened against the floor^17^, dramatically decreased ambulation, and failed to respond with attentive movements when their cage was tapped. In addition, the rats display Pica, the consumption of non-nutritive substances such as wood shavings^16,49^.

The IP LiCl injections were given around noon, approximately three hours after rats were fed. Finally, 48 h later, we lowered the electrodes ~ 300 μm into the pIC to perform a second experiment involving a LiCl injection.

LOB and Pica behaviors were evaluated in 17 experiments performed in nine rats after IP LiCl injection. In the ninth rat, we considered only the first LiCl experiment, since the others were prohibitively noisy.

The animals' behaviors were videotaped (Canon ZR45) during the experiments. We observed LOB and Pica for 1 h after lithium administration. Video images were analyzed every 30 s to quantify the occurrence, latency and time duration of both behaviors. Thus, the behavior was scored, and the timestamps of interest were extracted. While the first author of this paper was not blind to the NaCl and LiCl condition, the second coder was blind to these conditions. Both authors together manually scored LOB and Pica. Then and independently of the behavior scoring, the pIC neural activity was analyzed.

### Electrical microstimulation

Trains of biphasic square pulses (phase duration of 1 ms) lasting two minutes at 5, 10, 15, or 30 Hz and current intensity at 6 to 24 μA in steps of 2 uA were used (Multichannel Systems STG4008). During each session of stimulation, trains (trials) at different intensities of current for one specific frequency were applied through two wires of tetrodes that were used previously to record single units during LiCl experiments. On average, each animal received two daily sessions of four trials of stimulation. We estimated the number of successful sessions regarding the expression of LOB and Pica for each frequency, considering a session successful when one of these behaviors was observed in at least one of the four trials. We observed and videotaped the behavior for forty-five minutes after the beginning of the first train of pulses.

### Statistical analysis

We compared the LiCl and NaCl groups in terms of the time and latency of LOB and Pica behaviors using a one-tailed Mann–Whitney test given data did not fit a Gaussian distribution^50^. We rejected a null hypothesis of no difference on duration and latency between groups with a *p* value < 0.05.

To discriminate between putative pyramidal cells and interneurons, we evaluated the bi-modality of action potential width distribution (peak to trough durations) using the Hartigan's dip test^51^. We rejected a null hypothesis assuming a unimodal waveform distribution on width with a *p* value < 0.05.

To represent the magnitude of the FR changes, we split the whole recording period of 70 min into 140 bins, consisting of the 10 min before and 60 min after injection. A Z-score for each of these time bins was calculated, by comparing to the first 20 pre-injection bins^52^. To investigate the relationships among neurons placed around the same or different tetrodes, we computed the Pearson correlation coefficients (PCCs) between all possible pairs of the neurons from a same experiment for the total 17 experiments. Also, to study the oscillatory activity in single unit recordings, we computed the auto-correlograms for each neuron using a time window of + /− 1 s at a 1 ms temporal resolution. We computed the auto-correlogram every 30 s bin during the pre-injection, LOB, Pica and in-between LOBs periods. The auto-correlograms were averaged over the bins for each condition mentioned above. Then we calculated the power spectrum of these auto-correlograms. We also computed the power spectrum of LFP signals using the short-time Fourier transform method for the same periods as above using the electrodes that were also used to micro-stimulate the pIC.

We performed a statistical test to evaluate the FR changes of pIC neurons during LOB or Pica behaviors in response to LiCl administration. Our null hypothesis assumed that the FR remained unchanged regardless of behavior. In this test, we calculated the mean FR of neurons every 30 s. The test statistic was defined as the difference of the FRs between during the presence and the absence of a particular behavior. We performed a non-parametric test as described below, and elsewhere^53^, rejecting the null hypothesis with a *p* value < 0.01. To estimate the empirical distribution of the test statistic under the null hypothesis, we randomly mixed the FRs of LOB and non-LOB and then computed the difference of the average FRs in the same manner. The validity of this assumption stems from the null hypothesis that the FRs of both conditions were from the same distribution. We repeated this shuffling procedure 1000 times for each neuron. By comparing the test statistic computed using the original data to the estimated empirical distribution, we calculated the *p* value and rejected the null hypothesis when the test statistic was less than a significance level of 0.01. The same statistical tests were also performed for Pica and non-behavior episodes.

Neurons were classified as either LOB or Pica type if their FRs significantly changed during one of these behaviors, as having a generalized response if their FRs significantly changed during both of these behaviors and the non-behavior time, and as a mixed response if the FR changed during LOB but showed an opposite change during Pica and/or the non-behavior time. Differences in the proportions of neuronal types for LiCl and NaCl groups were evaluated using a one-tailed Fisher exact test. We reject the null hypothesis of same proportion of neurons in both groups when *p* value was < 0.05.

To better understand the prediction of the LOB binary outcome (or Pica) by the frequency of the electrical micro-stimulation pulses applied to the pIC, a logistic regression model was used on the whole set of trials (N = 121; 8 rats). We used a Wald test for assessing the significance of this variable predictor on the behavioral outcome of LOB. We rejected the null hypothesis of no correlation between stimulation frequency and LOB outcome with a *p* value < 0.05. The Pica data set was analyzed in a commensurate manner. Putative differences in the current magnitudes between the behavioral outcome and the frequencies of EMS were evaluated using a 2-way ANOVA test. We rejected the null hypothesis of no difference in the magnitude of current for a *p* value < 0.05.

### Histology

To verify the position of the electrodes, electrolytic lesions were created at the end of the last recording session by applying an anodic current of 25 µA for 20 s through two of the wires of each tetrode. Forty-eight hours after the electrolytic lesion, the animals were deeply anesthetized with chloral hydrate (7%; 350 mg/kg) and were perfused through their left ventricles with 500 ml of saline. This was followed by 500 ml of 10% neutralized formalin. The brain of each rat was then removed, post-fixed for 2 h in the same solution, and transferred to 30% sucrose in buffered phosphate saline with 0.02% sodium azide for 2 days. Each brain was then cut into 50 µm coronal series (freezing microtome), mounted on gelatinized slides, Nissl stained with cresyl violet and examined via light microscopy.

## Supplementary information

Supplementary Information.
