# Supplementary material for: Interoceptive insular cortex participates in sensory processing of gastrointestinal malaise and associated behaviors
Source: Sci Rep. 2020 Dec 10;10:21642. doi: 10.1038/s41598-020-78200-w (PMC7730439; doi:10.1038/s41598-020-78200-w)
Supplement: Supplementary file 1 — Supplementary Information. [file 41598_2020_78200_MOESM1_ESM.pdf]

Interoceptive insular cortex participates in sensory processing of gastrointestinal malaise and associated behaviors

Marcelo Aguilar-Rivera[1*], Sanggyun Kim[2], Todd Coleman[1], Pedro E. Maldonado[3,4+], and Fernando Torrealba[5+]

[+]Equal senior authors

(1) Department of Bioengineering. University of California, San Diego. La Jolla, 92093, USA.
(2) Psychiatry and Behavioral Sciences. Stanford University. Palo Alto, 94304, USA.
(3) Facultad de Medicina, Universidad de Chile. Santiago, 8380453, Chile.
(4) Biomedical Neuroscience Institute. Universidad de Chile. Chile.
(5) Departamento de Ciencias Fisiológicas. Pontificia Universidad Católica de Chile. Santiago, 8331150, Chile.

*Corresponding Author:

Dr. Marcelo Aguilar-Rivera

E-mail: mia003@eng.ucsd.edu

Supplemental Materials

Behavioral and neural response to IP NaCl injections. We studied LOB after NaCl administration in five out of the nine rats that also received an IP injection of LiCl in a subsequent experiment. We found that four out of five rats displayed a delayed latency and shorter periods of LOB after NaCl administration in comparison to IP LiCl injection (Figure supplementary 1A-B; Mann-Whitney test; $p < 0.05$). We did not observe Pica after the NaCl administration, or the facial expressions accompanying LOB following LiCl administration. These results demonstrate that the IP LiCl injections trigger a distinctive malaise, which is followed by stereotypical behaviors. In addition, the magnitude and proportion of pIC neurons responding to IP NaCl injection was smaller in comparison to LiCl administration, as shown and discussed in the main text along with Supplementary Table 1.

Recordings from the Secondary Somatosensory Cortex (SS2). To determine the specificity of pIC neurons, we compared their activity to the neuronal response of the SS2 for LiCl-induced malaise. We recorded 18 SS2 neurons from two additional rats that received IP injections of LiCl (Figure supplementary 2A). Only one neuron increased its activity during LOB. We found an increment of FR in close temporal association with the LiCl administration in fifty percent of SS2 neurons (9/18). However, none of the SS2 neurons showed sustained excitation over time (Figure supplementary 2B), unlike the pIC neurons that showed an increase in their FR in close temporal proximity to the LiCl injection. In five of the nine SS2 neurons that increased their FR around the time of injection, the increase was observed immediately after injection but dropped minutes later (Figure supplementary 2), suggesting it could be related exclusively to the manipulation/injection. This temporal pattern was not observed in pIC neurons. In the remaining four SS2 neurons, the increments in FR were also recorded between LOBs, and in two cases coincident with Pica epochs. These results demonstrate that only pIC neurons exhibit specific changes in activity related to the malaise triggered by the IP LiCl injections.

Electrophysiological and anatomical considerations. Neurons recorded from the same tetrode showed differences in the timing of their responses associated with LOB epochs (Figure supplementary 3A). We observed more than one type of change in FR per electrode in response to IP LiCl injection (Figure S3B-C). These findings suggest that neighboring neurons in pIC do not necessarily exhibit similar response properties, which occurs in other early

sensory cortices[1]. Moreover, there were different degrees of correlation between pairs of neurons recorded from the same electrode (Figure supplementary 3D-F). Fifteen percent of these pairs (17/113) showed a negative and significant correlation, and this proportion was larger than that of the pairs of neurons recorded with different tetrodes (Fisher exact test; $p < 0.0458$). On the other hand, we observed a larger proportion of pairs of neurons that showed a significant and positive correlation recorded from different tetrodes in comparison to those recorded from the same tetrode (Fisher exact test; $p < 0.0354$). This kind of push-pull mechanism could increase selectivity to interoceptive information, as has been described in other cortices[2]. For the pairs that were recorded from different electrodes, the distances between them ranged from 350 to 750 μm.

Furthermore, we found no evidence of spatial grouping of neurons with similar responses to GI malaise or its relief in the rostral-caudal axis of the pIC (Figure supplementary 4). In addition, electrical micro-stimulation applied at any level of the posterior IC triggered the behaviors of LOB and Pica (Figure S5). These results strongly suggest that neighboring neurons around the same electrode may respond differently to systemic LiCl administration, and thus do not necessarily share similar response properties.

References

1. Romo, R., Hernández, A., Zainos, A., Brody, C. D. & Lemus, L. Sensing without touching: Psychophysical performance based on cortical microstimulation. *Neuron* (2000). doi:10.1016/S0896-6273(00)81156-3
2. Priebe, N. J. & Ferster, D. Direction selectivity of excitation and inhibition in simple cells of the cat primary visual cortex. *Neuron* (2005). doi:10.1016/j.neuron.2004.12.024

Figure supplementary 1. Behavioral pattern triggered by IP NaCl injections is different from LOB expression triggered by LiCl administration. Rats exhibited LOB behavior after NaCl administration, however with delayed onset and shorter duration in comparison to IP LiCl injection. Bars represent median and whiskers the interquartile range.

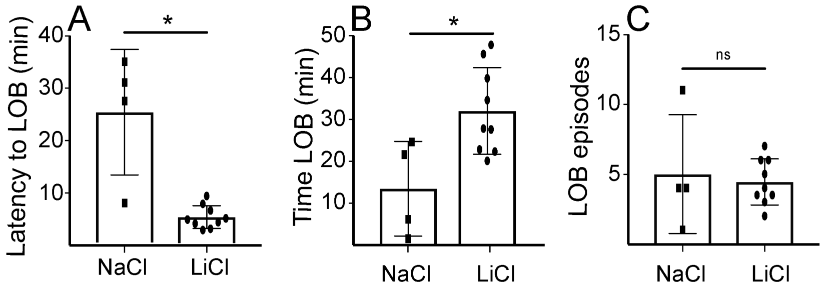

Supplementary Table 1. Comparison of FR responses to NaCl or LiCl injections.

Table S1. Comparison of firing rate responses to NaCl or LiCl injections.

Response types	Number and percentage of total		
	NaCl	LiCl	P Value
Increase with LOB	1 (3.3)	8 (24.2)	0.019
Decrease with LOB	2 (6.7)	3 (9.1)	0.546
Increase with pica	-	3 (9.1)	-
Mixed	-	5 (15.2)	-
Increase not associated	3 (10)	4 (12.1)	0.554
Decrease not associated	5 (16.7)	2 (6.1)	0.187
No change	19 (63.3)	8 (24.2)	0.001
Total	30 (100)	33 (100)	

*P values from Fisher exact test

Figure supplementary 2. Discharge patterns of SS2 neurons after LiCl administration. A. IP lithium injection-related spiking for all neurons. Many cells displayed increases in FR immediately after lithium administration, while a few of them showed increases at the end of the recording time. Vertical line depicts injection time. The arrow indicates the position in the spectrogram of the unit shown below. B. This neuron (basal mean FR of 9 spikes/sec) increased its activity only in response to the injection of LiCl. Dark and light gray shadows represent LOB and Pica respectively.

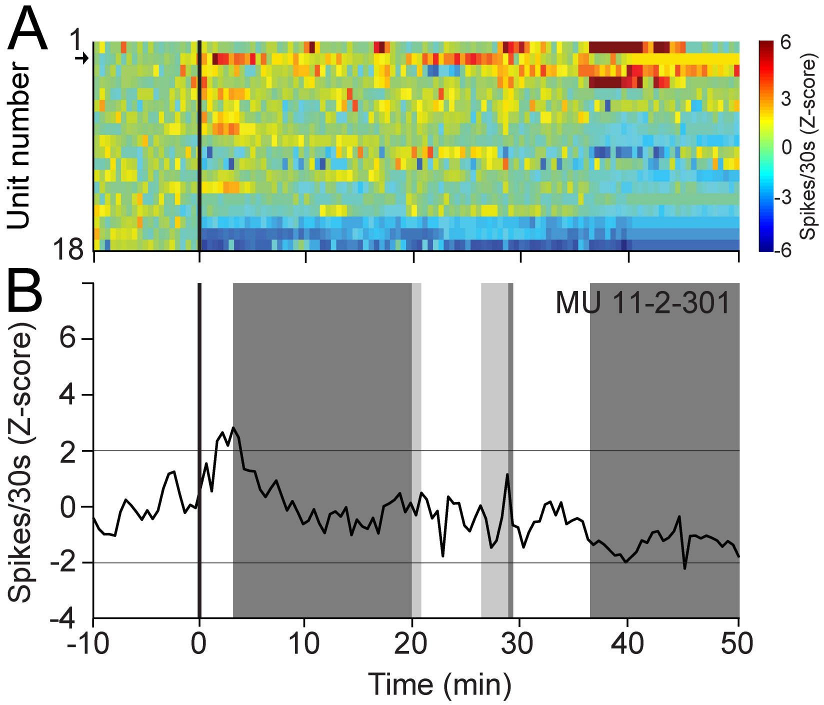

Figure supplementary 3. Neighboring pIC neurons do not exhibit similar response properties. A. The responses of three neurons after LiCl injection at time 0 and during three LOB episodes (dark gray) revealed that neurons #1 and #2 increased their FR during LOB, while neuron #3 showed a decrease in its FR that was not associated with LOB. B. Average waveform of the spikes from the three neurons shown in A, recorded by the four channels of the same tetrode. C. Most of the electrodes recorded neurons that showed two or more types of responses in relation to the behaviors triggered by LiCl administration. Symbol ♯ represents the number of rats where we identified these response types. D. Scatter-plot of cross-correlation coefficients among all the pairs of neurons recorded in each experiment with the same or different tetrodes. Pairs of neurons that showed a significant correlation ($p < 0.01$) are represented in bold. E. More pairs of neurons recorded with the same tetrode showed a negative and significant correlation, while more pairs of neurons recorded with different tetrodes showed a positive and significant correlation. F. Upper charts are examples of FR changes of pairs of neurons that showed a strong positive correlation and were recorded from different tetrodes. The two lower pairs are examples of neurons that showed a strong negative correlation and were recorded with the same tetrode. These four examples of pairs of neurons are those represented by asterisks at the top right and bottom left in D. Dark and light gray shadows represent LOB and Pica respectively.

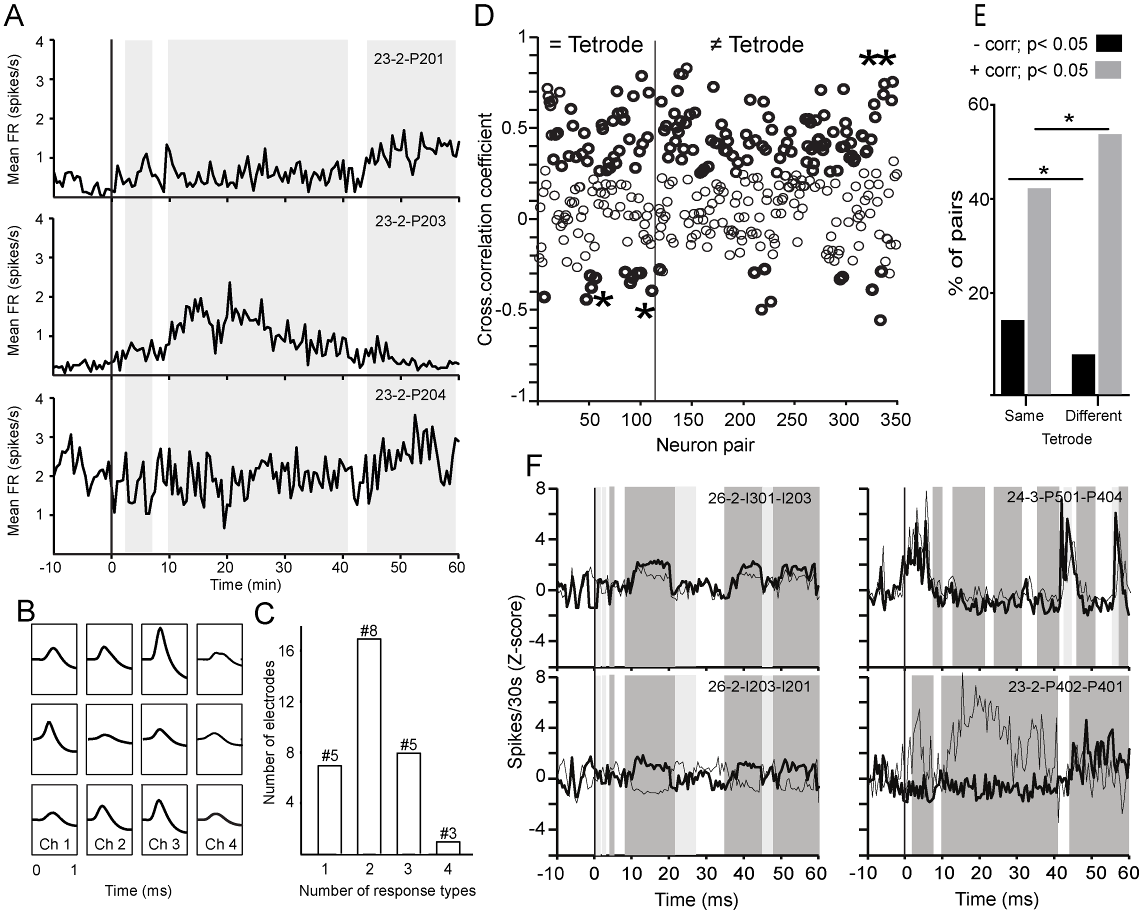

Figure supplementary 4. Schematic distribution of all neurons in the rostro-caudal axis of the pIC that responded to GI malaise. Neurons were drawn on representative coronal sections from the electrode position for all the rats. Symbol ♯ represents the number of rats studied in each level in the electrophysiological experiments. Bold symbol shows levels from bregma where electrical stimulation was applied triggering the behaviors of LOB and Pica. The histological analysis suggests that most of the neurons (81/105) in the nine rats were recorded between layers four and six of the IC, and that the recorded area extended from +1.4 to -2 mm relative to bregma.

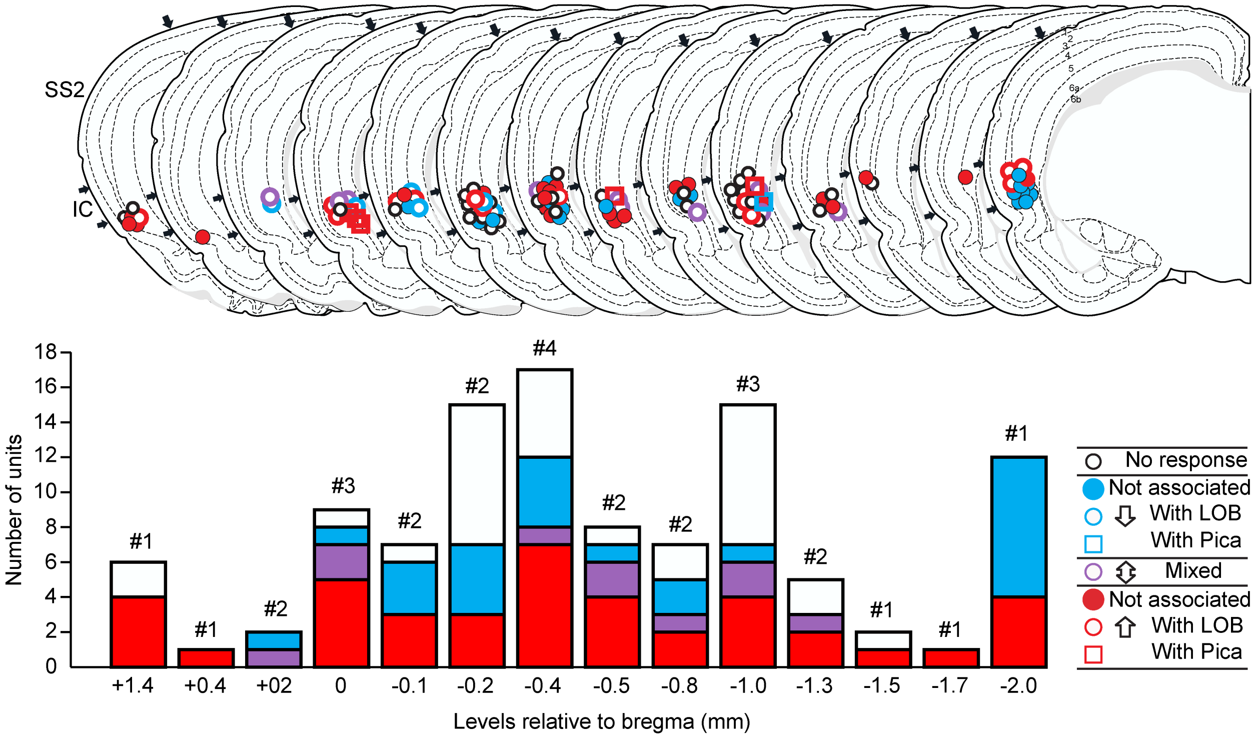

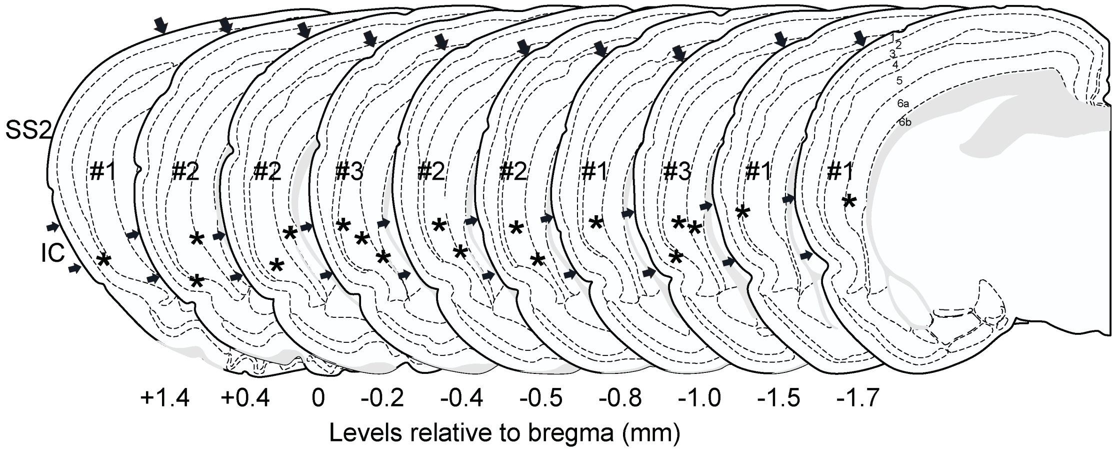

Figure supplementary 5. Depiction of the rostro-caudal pIC sites where EMS triggered LOB and Pica behaviors. Integer adjacent to symbol ♯ represents the number of rats in which EMS triggered both behaviors. Asterisk depicts the mark gotten by electrolytic lesion corresponding to the electrode tip. We found no evidence of a particular locus for LOB and Pica.
